# Supplementary figures and images for: The weekend effect in kidney transplantation outcomes: A meta-analysis
Source: PLoS One. 2023 Jun 16;18(6):e0287447. doi: 10.1371/journal.pone.0287447 (PMC10275449; doi:10.1371/journal.pone.0287447)

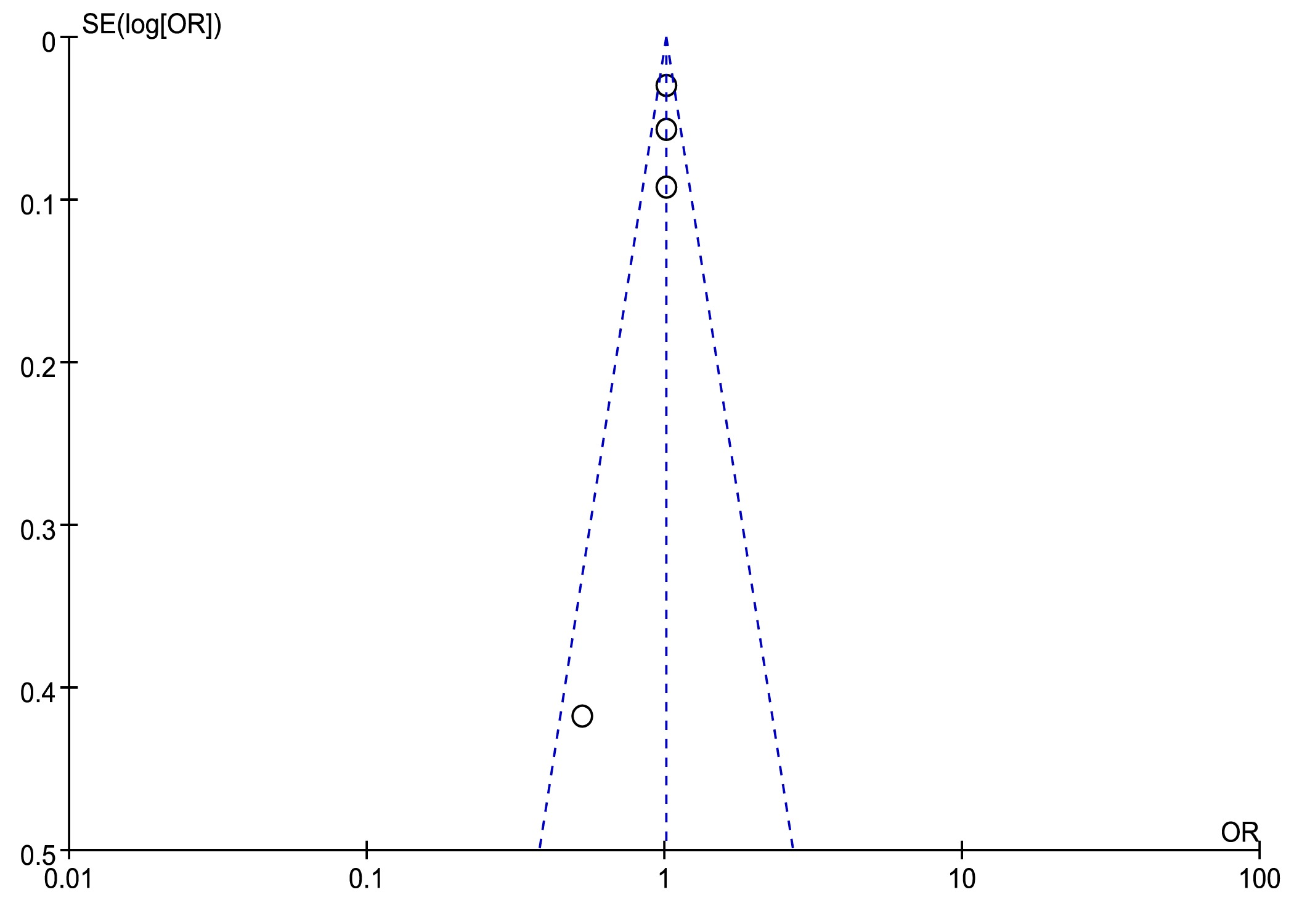

Supplement: S1 Fig — (TIF) [file pone.0287447.s001.tif]
