# Supplementary material for: The weekend effect in kidney transplantation outcomes: A meta-analysis
Source: PLoS One. 2023 Jun 16;18(6):e0287447. doi: 10.1371/journal.pone.0287447 (PMC10275449; doi:10.1371/journal.pone.0287447)
Supplement: Attachment — Submitted filename: Response to Reviewers.docx [file pone.0287447.s003.docx]

Dear Editors and Reviewers:

Thank you for your letter and for the reviewers’ comments concerning our manuscript entitled “The weekend effect in kidney transplantation outcomes: a meta-analysis” (ID:PONE-D-23-05474). Those comments are all valuable and very helpful for revising and improving our paper. We have studied comments carefully and have made correction which we hope meet with approval. Revised portion are marked in yellow in the paper. The main corrections in the paper and the responds to Reviewer’s comments are as following:

1. Response to Reviewer #1 :

We appreciate for your warm work earnestly, and hope that the correction will meet with approval. We explored that there was no difference in survival rates between the graft and the recipient, and our discussion of standardized procedures and decision algorithms for the transplant process was clear. In addition, we discussed the experience of transplant surgeons during weekend surgeries and their impact on early surgical complications and transplant outcomes. Similarly, although there are different service systems in different transplant centers, always there is some system of duty-on-call, allowing to coordinate the presence of licensed transplant surgeon during week-ends, to perform the procedure, effectively avoiding the occurrence of transplant complications. We apologize that the quality of our research is limited, but our research is meaningful. We will further update the latest research, although the statistical methods used in the study can be replaced by artificial intelligence. In addition, we further explored the significance of early surgical complications on early and late kidney transplant function and survival.

2. Response to Reviewer #2 :

Thank you very much for appreciating my article. The literature for meta-analysis comes from databases available online. If access to some restricted databases is available, the number of surgeries included in the literature will increase. Of course, better results may be possible, and we will also update the evaluation in the future. Secondly, we also added in the discussion that "if a kidney transplant is performed in the middle of the night, the surgeon may not be able to concentrate due to fatigue, which may be related to the better effect of the surgery at the beginning of the day on weekends, but the overall results of the transplant on weekends and weekdays are the same." We fully agree with this valuable opinion.

3. Response to Reviewer #3 :

Most deceased-donor kidney transplants are emergency operations, and the time point of transplantation depends on the time point of kidney acquisition. The cold ischemia time of most deceased-donor kidneys is around 5-10 hours, and a longer cold ischemia time can lead to an increase in the proportion of delayed renal function recovery after transplantation.

We tried our best to improve the manuscript and made some changes in the manuscript to meet publication criteria. These changes will not influence the content and framework of the paper. And here we did not list the changes but highlighted in yellow in revised paper.

Once again, thank you very much for your comments and suggestions.

Yours sincerely,

Xiaoli Huang

Email: 973229194@qq.com
